# NURR1 deficiency is associated to ADHD-like phenotypes in mice

**DOI:** 10.1038/s41398-019-0544-0

**Published:** 2019-08-27

**Authors:** Francesca Montarolo, Serena Martire, Simona Perga, Michela Spadaro, Irene Brescia, Sarah Allegra, Silvia De Francia, Antonio Bertolotto

**Affiliations:** 1 Neuroscience Institute Cavalieri Ottolenghi (NICO) Orbassano (Turin), Orbassano, Italy; 2 Neurobiology Unit, Neurology—CReSM (Regional Referring Center of Multiple Sclerosis), AOU San Luigi Gonzaga Orbassano (Turin), Orbassano, Italy; 3 Department of Neuroscience “Rita Levi Montalcini”, University of Turin, Turin, Italy; 4 Department of Biological and Clinical Sciences, University of Turin, AOU San Luigi Gonzaga Orbassano (Turin), Turin, Italy

**Keywords:** Neuroscience, ADHD

## Abstract

The transcription factor NURR1 regulates the dopamine (DA) signaling pathway and exerts a critical role in the development of midbrain dopaminergic neurons (mDA). NURR1 alterations have been linked to DA-associated brain disorders, such as Parkinson’s disease and schizophrenia. However, the association between NURR1 defects and the attention-deficit hyperactivity disorder (ADHD), a DA-associated brain disease characterized by hyperactivity, impulsivity and inattention, has never been demonstrated. To date, a comprehensive murine model of ADHD truly reflecting the whole complex human psychiatric disorder still does not exist. NURR1-knockout (NURR1-KO) mice have been reported to exhibit increased spontaneous locomotor activity, but their complete characterization is still lacking. In the present study a wide-ranging test battery was used to perform a comprehensive analysis of the behavioral phenotype of the male NURR1-KO mice. As a result, their hyperactive phenotype was confirmed, while their impulsive behavior was reported for the first time. On the other hand, no anxiety and alterations in motor coordination, sociability and memory were observed. Also, the number of mDA expressing tyrosine hydroxylase, a rate-limiting enzyme of catecholamines biosynthesis, and DA level in brain were not impaired in NURR1-KO mice. Finally, hyperactivity has been shown to be recovered by treatment with methylphenidate, the first line psychostimulant drug used for ADHD. Overall, our study suggests that the NURR1 deficient male mouse may be a satisfactory model to study some ADHD behavioral phenotypes and to test the clinical efficacy of potential therapeutic agents.

## Introduction

The nuclear receptor related 1 protein (NURR1, also called NR4A2) is an orphan member of the steroid hormone receptor family. It is a transcription factor essential for the development and functioning of the dopaminergic circuitry, particularly of midbrain dopaminergic neurons (mDA). NURR1 is required for mDA generation, as its ablation leads to their full agenesis^1,2^. It also plays a critical role in the migration and target area innervation of differentiating mDA in the striatum^3^. In mature mDA, NURR1 regulates genes of the dopamine (DA) signaling pathway, including tyrosine hydroxylase (TH), DA transporter 1 (DAT1), and vesicular monoamine transporter 2 (VMAT2)^2,4,5^. Finally, NURR1 exerts an anti-inflammatory function in microglia, which protects mDA from inflammation-induced death^6,7^. Given its crucial functions, altered NURR1 expression is implicated in DA-associated brain disorders, including Parkinson’s disease (PD)^8,9^ and schizophrenia^10,11^.

ADHD is an heterogeneous developmental brain disorder, which affects children but often leads to adverse consequences in adulthood, including drug abuse, delinquency, anxiety, depression, and social rejection^12,13^. It is characterized by three main symptoms, namely hyperactivity, impulsivity and inattention. Methylphenidate (MPH), with a history of use spanning ~5 decades, is the first line psychostimulant treatment for ADHD. MPH enhance DA transmission through multiple actions, including blockade of the DA reuptake transporter and amplification of the DA response duration^14^. Overall, changes in catecholaminergic tone induced by the chronic treatment of MPH lead to an improvement of the ADHD symptoms, although not without side effects, such as fatigue, nausea, and loss of appetite^15^.

It is generally accepted that ADHD has a strong genetic component^13^, although genome-wide association studies (GWASs) published to date are far from being conclusive, mostly due to their small sample size^16,17^. Single nucleotide polymorphisms in the NURR1 gene have been studied in humans ADHD^18^. However, the association between NURR1 polymorphism and gene expression has been only found in in vitro experiments and not directly in human ADHD^18^. Furthermore, the constitutive deletion of NURR1 in mice have been reported to increase spontaneous locomotor activity, a phenotype characteristic of the ADHD symptomatology^19–22^. However, it is still not clear whether the NURR1-knockout (NURR1-KO) mice can be considered as a comprehensive model of ADHD. Ideally, a comprehensive animal model should (I) mimic the fundamental behavioral characteristics of ADHD (face validity), (II) involve a similar pathophysiological mechanism (construct validity), and (III) predict responses to medications that could be used in ADHD treatment (predictive validity). To date, hyperactivity is still the only ADHD-like symptom described in NURR1-KO mice. Impulsivity has never been examined, while attention has been evaluated in a single study, which did not highlight an altered behavior^23^.

Although brain abnormalities, especially in the regions of basal ganglia, prefrontal cortex, and cerebellum, have been documented in patients with ADHD^24,25^, the exact pathogenesis of the disease remains elusive. A deeper knowledge of ADHD neuroanatomy is necessary to improve treatments, and reliable animal models are crucial to achieve it. Besides NURR1-KO mice, some murine models have been proposed as ADHD models, mostly based on targeting genes involved in DA transmission^26,27^.

Here we examined the effects of the constitutive deletion of NURR1 on the behavior and the number of TH-expressing mDA of adult male mice. We focused on locomotor activity, and impulsivity, but for an exhaustive characterization we also evaluated motor coordination, anxiety, sociability and memory. Finally, we examined whether an altered behavioral phenotype could be recovered following the exposition to human pharmacological doses of MPH.

## Materials and methods

### Animals

Three to five months old male NURR1-knockout mice (NURR1-KO, *n* = 64) and their wild-type (WT, *n* = 68) littermates were used for all experimental paradigms (see Supplementary Materials, Table S1). Specifically, one cohort of 9 WT and 7 NURR1-KO untreated mice underwent the behavioral tests. At the end of the behavioral tests, these mice were sacrificed to perform the histological procedures. A second cohort of 7 WT and 4 NURR1-KO untreated mice were used for biomolecular analysis. A third cohort of 6 WT and 10 NURR1-KO were used for blood pressure, heart rate and DA measurement in brain and plasma. A fourth and fifth cohort comprising 42 WT and 47 NURR1-KO mice were used for methylphenidate experiments. The NURR1-KO mice were obtained from Prof. Orla M. Conneely, Baylor College of Medicine, Houston, USA. Since homozygous NURR1-KO mice die within 12 h (h) after birth^2,28^, heterozygous mice were used. Their genotype were confirmed by means of polymerase chain reaction (PCR)^28^. All experimental procedures were carried out at NICO, approved by the Ethical Committee of the University of Torino and authorized by the Italian Ministry of Health (authorization number: 56/2017-PR). The experiments have been carried out in accordance with the European Communities Parliament and Council Directives of 24 November 1986 (86/609/EEC) and 22 September 2010 (2010/63/EU). Mice were housed with a 12 h light/dark cycle and free access to food/water. Adequate measures were taken to minimize pain and discomfort.

### Behavioral tests

The WT and NURR1-KO animals underwent the behavioral tests always during the light phase of the cycle, leaving at least a week of break between tests. The experiments were performed under dim white light conditions (2 lux). At the end of each trial, the equipment were accurately cleaned with ethanol 2% and water. Where needed (i.e. open field; OF, elevated plus maze; EPM, Morris water maze, three chamber sociability), behavioral procedures were video-recorded and scored by an individual blind to the genotype of the mouse. Data were analyzed using Ethovision XT9 video track system (Noldus Information Technology, Wageningen, The Netherlands).

### Open field test

Locomotor activity was investigated by means of the Open field (OF) test. On test day, mice were transported to the testing room and left undisturbed for 1 h before testing. Each animal was placed in the corner of the arena (50 × 50 × 50 cm) for 1 h. Total and 10-min bins distance traveled, velocity and time spent in the central area (25 × 25 cm) were video-recorded.

### Cliff avoidance reaction test

Impulsivity was investigated by means of Cliff avoidance reaction (CAR) test. On test day, mice were transported to the testing room and left undisturbed for 1 h before testing. CAR was assessed^27^ using a round wooden platform (diameter 20 cm), supported by a rod (height 50 cm). The platform was secured so that the movement of the animal did not affect it. The floor below the platform was carpeted to prevent injury in case of a fall. The test was initiated by gently placing an animal on a platform such that the forelimbs approached its edge. Mice which fell from platforms were immediately and gently placed back on the platforms, and the test was continued until 1 h had elapsed. Mice, which did not fall from platforms, were tested for the same time period. The number of the mice which fell from platforms and the time of fell have been recorded and reported as percentage of the number of tested mice and time course of CAR impairment, respectively.

### Rota-rod test

Motor performance and coordination were investigated by means of rota-rod test. Mice were tested for three consecutive days. In each day, after a 2 min (min) training session at a constant speed (4 rpm), the mice received three test sessions (T) in which the rod (Mouse Rota‐Rod, Ugo Basile Biological Research Apparatus, Comerio, Italy) accelerated continuously from 4 to 65 rpm over 350 s ^29^. The latency to fall off the rod was recorded.

### Elevated plus maze test

Anxiety-like behavior was investigated by means of Elevated plus maze (EPM) test. On test day, mice were transported to the testing room and left undisturbed for 1 h before testing. The EPM test apparatus was a plus-cross shaped constructed from gray forex raised 60 cm above the floor. It comprised two open arms (30 × 5 × 0.20 cm) and two closed arms (30 × 5 × 15 cm walls) originating from a central platform (5 × 5 cm). At the beginning of each trial each animal was gently placed in the center of the plus maze, facing an open arm and it was allowed to explore the maze for 5 min^30^. The number of entries in either open and closed arms and their respective cumulative time spent was video-recorded and reported as a frequency of entries and time spent in the open arms. An animal was considered to have entered an arm of the plus maze when all four paws had left the central platform.

### Morris water maze test

Spatial learning and memory of mice were investigated by means of Morris water maze test (see Supplementary Materials)^31–33^.

### Three-chambered sociability test

On test day, mice were transported to the testing room and left undisturbed for 1 h before testing. The test was designed as previously described^34,35^ (see Supplementary Materials).

### Histological procedures

Seven NURR1-KO and nine WT littermates mice were deeply anesthetized (ketamine 200 mg/kg, xylazine 50 mg/kg) and trans-cardially perfused with 4% paraformaldehyde in 0.12 M phosphate buffer, pH 7.2–7.4. The brains were removed and immersed in the same fixative at 4 °C for 24 h and then cryo-protected in 30% sucrose in 0.12 M phosphate buffer. Brains were frozen and serially cut by a cryostat in 30 µm-thick coronal sections collected in phosphate buffered saline (PBS). In order to detect tyrosine hydroxylase (TH) mDA neurons, slices were incubated overnight at 4 °C with the monoclonal anti-mouse TH (22941, Immunostar, Hudson, WI, USA) diluted 1:1000 in PBS with 0.25% Triton X-100 and 1.5% normal goat serum. Immunohistochemical reactions were performed by the avidin–biotin–peroxidase method (Vectastain ABC Elite kit; Vector Laboratories, Burlingame, CA, USA) and revealed using 3,3′-diaminobenzidine (3% in Tris–HCl) as chromogen. Images for morphometric analysis were acquired by means of the Nikon Eclipse E600 microscope equipped with the Neurolucida system (Micro Bright Field, Williston, VT, USA) and analyzed by means of the ImageJ software (http://rsbweb.nih.gov/ij/index.html). TH positive ( + ) cell number (cell number/mm^2^) in the whole substantia nigra (SN) and ventral tegmental area (VTA) (Bregma −2.92 mm, Interaural 0.88; Bregma −3.88 mm, Interaural −0.08) were quantified. At least three sections for animal were evaluated.

### Biomolecular analysis

Four NURR1-KO and seven WT littermates mice were euthanized by inhalation of isoflurane and brains were removed. The brain areas (i.e. SN and VTA) were manually dissected from 300 µm-thick coronal sections cutter by vibratome (Bregma −2.92 mm, Interaural 0.88; Bregma −3.88 mm, Interaural −0.08). All samples were rapidly frozen in 2-methylbutane in dry ice and total RNA was isolated by extraction with the Pure Link RNA Mini Kit (Thermo Fisher Scientific, US) according to the manufacturer’s instructions. Total RNA was reverse-transcribed to complementary DNA (cDNA) at a final concentration of 20 ng/μl using the High Capacity Kit (Thermos Fisher Scientific, Waltham, MA, USA). Gene expression analysis was performed by real-time PCR using Applied Biosystems’ TaqMan gene expression products (Thermos Fisher Scientific, Waltham, MA, USA). Transcriptional expression was normalized using glyceraldehyde-3-phosphate dehydrogenase (GAPDH) as reference gene. For primers and probes, Applied Biosystems’ TaqMan® Assay-on-demand-TM gene expression products were used (GAPDH; Mm99999915_g1, TH; Mm00447557_m1). Expression levels of target genes were calculated by the normalized comparative cycle threshold (Ct) method (2^-ΔCt^).

### DA isolation and measurement

Ten NURR1-KO and six WT littermates mice were euthanized by inhalation of isoflurane and brains were removed and blood was collected into EDTA tubes. The brains were rapidly frozen in 2-methylbutane in dry ice. Blood samples were centrifuged 10 min at 3000 G at 4 °C. The plasma was removed and rapidly frozen in 2-methylbutane in dry ice. Brains and plasma were stored at −80 °C until use.

DA-1,1,2,2-d4 hydrochloride, trifluoroacetic acid and tetrahydrofuran for liquid chromatography were purchased from Sigma-Aldrich Corporation (Milan, Italy). Acetonitrile (HPLC grade) was purchased from VWR (Milan, Italy). HPLC-grade water was produced by a Milli-DI system coupled with a Synergy 185 system by Millipore (Milan, Italy). Previously weighted brain samples were frozen in liquid nitrogen, sonicated for 1 min, reconstituted in 1 mL of water and sonicated for another min. The calibration curve of DA was established in the concentration range of 300–100,000 ng/mL. Five hundred microliter plasma or brain samples were extracted by protein precipitation using 1 ml of acetonitrile/2-propanol. Each sample was vortexed for at least 15 s and then centrifuged at 12,000 rpm for 10 min. The eluates were evaporated with the Eppendorf AG Concentrator Plus (Eppendorf AG, Hamburg, Germany). The extracted sample was reconstituted with 250 μL of 97% of water (0.1% trifluoroacetic acid)/acetonitrile (90/10) and 3% of tetrahydrofuran and transferred to an injection vial. Chromatographic separation was performed at 35 °C, using a column oven, on a C18 reverse-phase column (LiChrocart 250-4 Lichrospher 100RP-18 5 um, VWR, Germany), protected by a Security Guard (VWR) precolumn. The mobile phase was composed 97% of water (0.1% trifluoroacetic acid)/acetonitrile (90/10) and 3% of tetrahydrofuran. The flow rate was set at 1 mL/min. DA plasma concentrations were reported as ng/mL, instead brain amount were converted in ng/mg of tissue weight.

### Methylphenidate administration

NURR1-KO and WT littermates were administered a saline solution (vehicle, 0.9% NaCl) of Methylphenidate (MPH, racemic mixture, Sigma-Aldrich, St. Louis, MO) or saline alone (0.9% NaCl, vehicle) by oral gavage using a feeding needle (Fisher Scientific, Pittsburgh, PA) (Italian Ministry of Health authorization number: SP/005). MPH was administered at 0.75 mg/kg dose 1 h before behavioral test as reported in ref. ^36^. This oral dose used has been demonstrated to produces serum levels of MPH equivalent to those seen in clinically treated humans^36^. We did not withhold food prior to the gavage. Mice were not anesthetized during the gavage procedure. To avoid confounding results due to the double administration of MPH, male NURR1-KO and WT mice never tested before for behavioral analysis and never treated before with MPH were treated with the drug or vehicle and randomized to underwent OF or CAR test as indicated above. Specifically, 13 WT treated with vehicle, 14 NURR1-KO treated with vehicle, nine WT treated with MPH, 10 NURR1-KO treated with MPH were tested in the OF, whereas 11 WT treated with vehicle, 13 NURR1-KO treated with vehicle, nine WT treated with MPH, 10 treated with NURR1-KO MPH tested in the CAR (Supplementary Materials, Table S1).

### Statistic

Power analysis has not been performed, due to the several outcomes that we planned to analyze in this study. Continuous data were presented as medians and ranges. Normality of distribution was assessed by the Shapiro-Wilk test and homogeneity of variance with Levene test. Fisher’s exact test was used to compare the number of fallen at the CAR test between groups. Two-tailed t-test and Mann–Whitney U test were used to compare continuous data between groups, as appropriate. Linear mixed effects models (LMM) were used to compare longitudinal data between groups. Kruskal–Wallis test with Dunn’s post hoc test was used to compare the travelled distance at the OF test after MPH treatment between groups. P values were adjusted for multiple comparisons using the Benjamini–Hochberg method to control the false discovery rate (FDR). Statistical significance was considered at *p* values < 0.05. All analyses were carried out using R version 3.5.1 (www.r-project.org).

## Results

### NURR1-KO mice exhibit hyperactivity and impulsivity

**Fig. 1:**
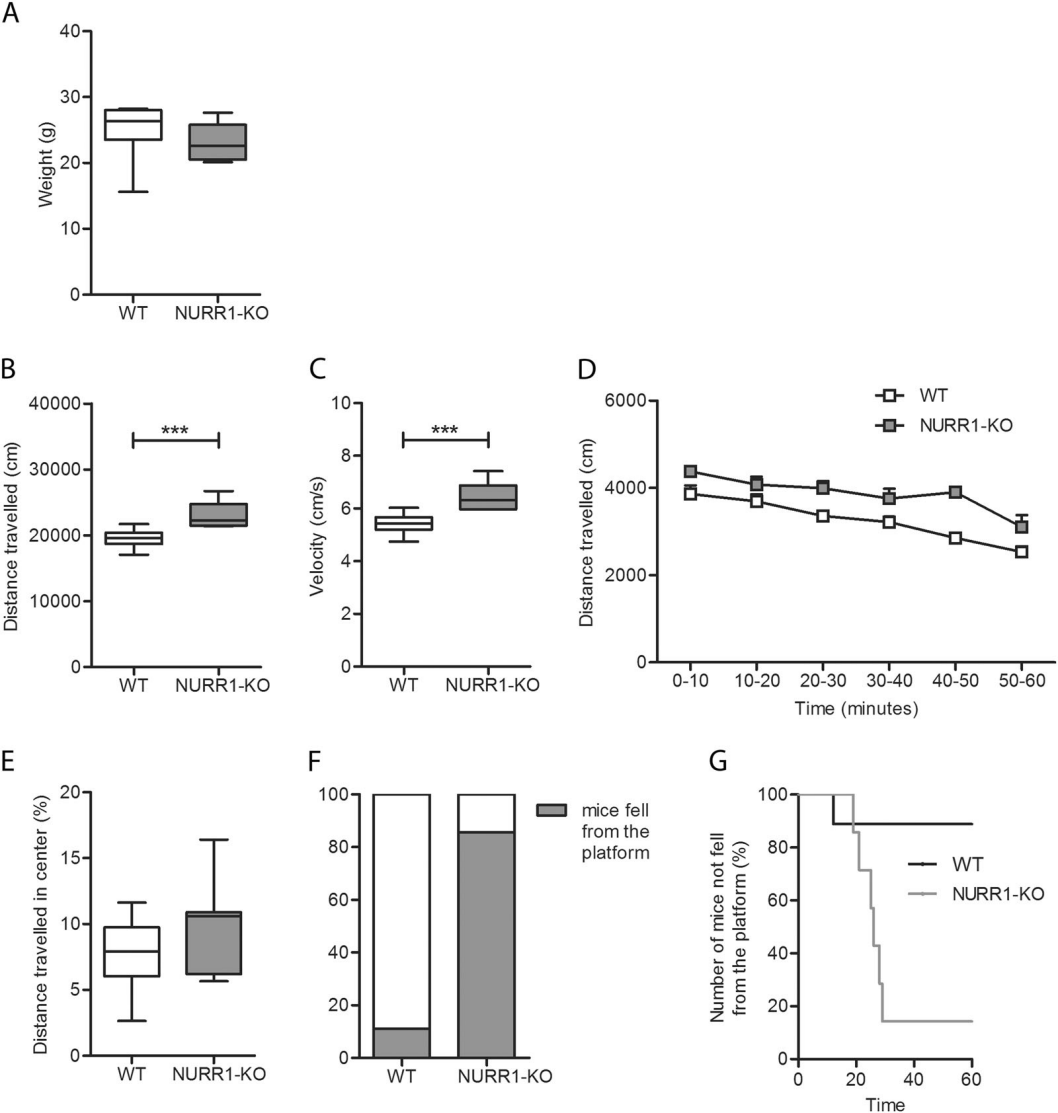
NURR1-KO mice show an increased locomotor activity and impulsivity. **a** Both WT (*n* = 9) and NURR1-KO (*n* = 7) mice were weighted before the tests. No major differences were detected in body weight between WT and NURR1-KO mice (Mann–Whitney U test, *p* = 0.07). Both WT (*n* = 9) and NURR1-KO (*n* = 7) mice were tested in the open field (OF, **b–e**) and cliff avoidance reaction test (CAR, **f-g**). **b**, **c** The OF analysis disclosed that NURR1-KO mice showed an increased distance travelled (*t*-test, *p* = 0.002) and velocity (*t*-test, *p* = 0.01) in comparison to their WT littermates. **d** The line plot shows distance moved (cm) as a function of 10-min bins, and the bar plot depicts the mean distance traveled (cm) per bin. Analysis revealed that genotype conditions (LMM, *p* = 0.0006) and time (LMM, *p* = 0.0001) significantly influences locomotor activity. **e** In the same behavioral test, NURR1-KO mice did not show an anxious phenotype indicated by the distance travelled in center compared with their WT littermates (*t*-test, *p* = 0.25). **f** The analysis of CAR test in WT and NURR1-KO mice displayed that the 85.7% of the NURR1-KO mice fell from platforms (gray box) during the 60 min observation against to the 11.1% of WT (Fisher exact test, *p* < 0.0001). **g** Time course of CAR impairment in WT and NURR1-KO mice was reported and about the totality (85.7%) of the NURR1-KO mice dropped from the platforms within 30 min

NURR1-KO mice developed normally with no major differences in body weights (Fig. 1a, Mann–Whitney U test, *p* = 0.07). Also, the systolic blood pressure and the heart rate were measured in WT and NURR1-KO mice highlighting no difference between genotypes (see Supplementary Materials, Figure S1A-B, Mann–Whitney U test, *p* = 0.44 for systolic blood pressure, *p* = 0.44 for heart rate). The spontaneous locomotor activity was measured by the OF test, evaluating the total distance traveled and the velocity. When placed in a novel open field, NURR1-KO mice resulted significantly more active compared to their WT littermates (Fig. 1b, t-test, *p* = 0.002; Fig. 1c, *t*-test, *p* = 0.01). The analysis revealed that locomotor activity is significantly affected by both genotype and time (Fig. 1d). Particularly, in the 10-min bins analysis, the distance travelled was overall greater in NURR1-KO mice compared to WT mice (LMM, *p* = 0.0006), while it significantly decreased over time in both groups (LMM, *p* = 0.0001). The analysis of the distance travelled in the center of the OF did not highlight an anxiety-like behavior of the NURR1-KO mice (Fig. 1e, *t*-test, *p* = 0.25).

The CAR test was performed to evaluate the impulsivity (Fig. 1f–g). As a result, 85.7% (six out of seven) of the NURR1-KO mice fell from platforms during the 60 min observation against the 11.1% (one out of nine) of the WT mice, thus showing an impulsive behavior (Fig. 1f, Fisher exact test, *p* < 0.0001). In addition, the time course of CAR impairment was reported indicating that about the totality (85.7%) of the NURR1-KO mice dropped from the platforms within 30 min (Fig. 1g).

### NURR1-KO mice do not show alterations in motor performance and coordination

**Fig. 2:**
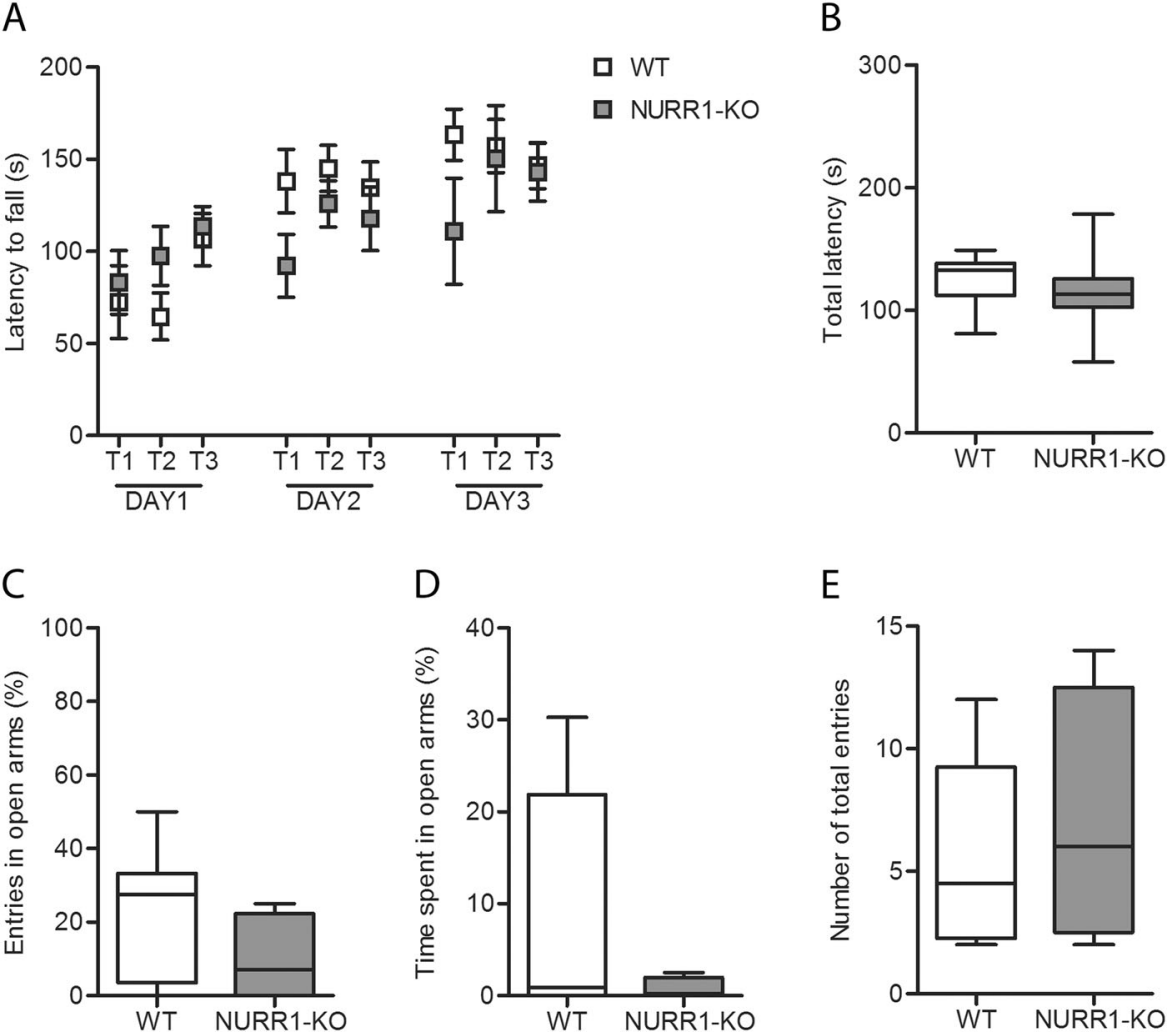
NURR1-KO mice do not show alteration in motor performance and coordination and anxiety-like behavior Both WT (*n* = 9) and NURR1-KO (*n* = 7) mice were tested on the rota-rod (**a**, **b**) and in the elevated plus maze (EPM) test for 5 min (**c–e**). **a** The analysis revealed that time conditions (LMM, *p* < 0.0001) but not genotype (LMM, *p* = 0.47) significantly influences motor performance. **b** No differences were detected in the total latency during the 3 days of trials (*t*-test, *p* = 0.51). **c**, **d** NURR1-KO did not reveal anxiety levels higher than WT mice analyzing the frequency of the entries in the open arm (**c** *t*-test, *p* = 0.16) and the percentage of the time spent in the open arm (**d** Mann–Whitney U test, *p* = 0.34). **e** No differences were detected also in the number of total entries (*t*-test, *p* = 0.66)

Motor coordination was evaluated using the accelerating rota-rod test (Fig. 2a, b). As demonstrated by the comparison of the latency to fall in the first trial of the first day (T1 DAY1) between the two groups, WT and NURR1-KO mice exhibited similar basal motor coordination (Fig. 2a, T1 Day 1). Subsequently, they both showed a significant improvement over days (LMM, *p* < 0.0001), independently from genotype (LMM, *p* = 0.47). Also the total latency during the 3 days of trials did not differ between the two groups of mice (Fig. 2b, *t*-test, *p* = 0.51).

### NURR1-KO mice do not show an anxiety-like behavior

**Fig. 3:**
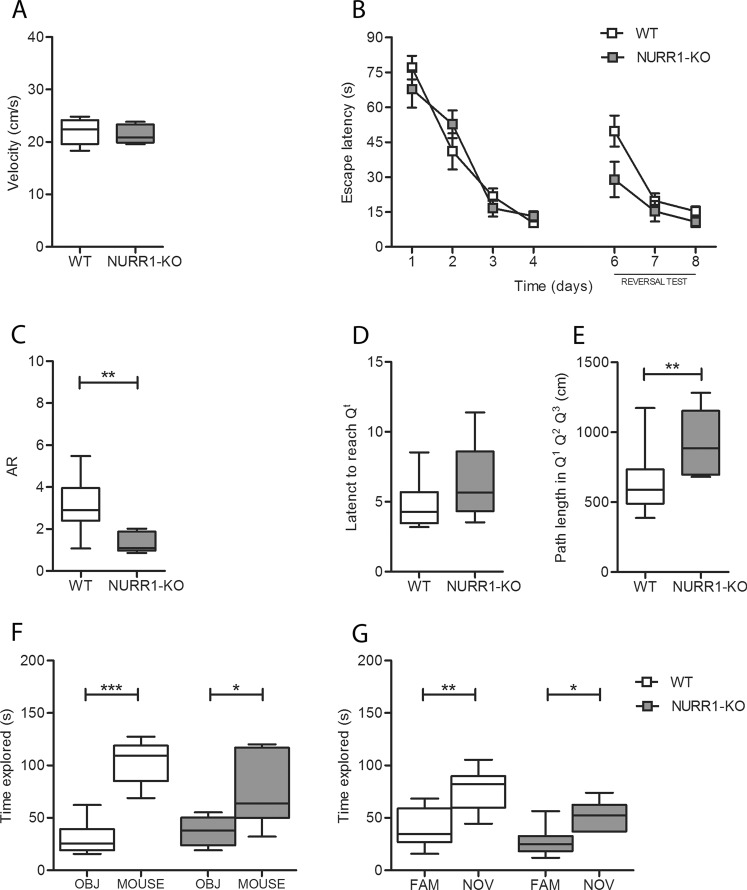
NURR1-KO mice do not show a defect in learning and memory. Both WT (*n* = 9) and NURR1-KO (*n* = 7) were tested in the Morris water maze (**a–e**) and three chamber maze test (**f**, **g**). **a** The basal level of the swimming velocity was not different between groups (*t*-test, *p* = 0.63). **b** Mice improved their performance across days without differences between genotypes. The analysis revealed that time conditions (LMM, *p* < 0.0001) but not genotype (LMM, *p* = 0.99) significantly influences spatial learning and memory. **c–e** During the probe trial NURR1-KO mice showed a reduce accuracy ratio (AR) in comparison to their WT littermates (**c**) (*t*-test, *p* = 0.002) without differences in the latency to reach the target quadrant (**d**) (Mann–Whitney U test, *p* = 0.11). **e** Measures of the total distance travelled (cm) after they reached the target zone in the probe trial showed longer path length outside the target zone (Q^1^, Q^2^, Q^3^) in NURR1-KO mice compared to WT mice (*t*-test, *p* = 0.03). **f** Mice displayed sociability, defined as spending more time sniffing the mouse than sniffing the object (*t*-test, WT: *p* < 0.0001, NURR1-KO: *p* = 0.03). **g** NURR1-KO mice do not show impairment in social memory spending more time sniffing the novel mouse than sniffing the familial one as WT (*t*-test, WT: *p* = 0.001, NURR1-KO: *p* = 0.01)

The EPM test was executed to confirm the absence of an anxiety-like behavior in NURR1-KO mice, suggested by the OF test (Fig. 1e). The frequency of entries (Fig. 2c, *t*-test, *p* = 0.16) and the time spent (Fig. 2d, Mann–Whitney U test, *p* = 0.34) in the open arms was similar in NURR1-KO mice and their WT littermates. Also the number of total entries did not differ between the two groups, indicating that the test was well-conducted (Fig. 3e, *t*-test, *p* = 0.66).

### NURR1-KO mice do not show a defect in spatial memory and learning

Spatial memory was tested by means of the Morris water maze (Fig. 3a–e). During the first trial of the first day (when the mice knew anything about the spatial location of the platform) there was no difference in the swimming velocity between groups (Fig. 3a, *t*-test, *p* = 0.63), suggesting normal vision, locomotor skills, and motivation in NURR1-KO animals. The acquisition phase of the test (Fig. 3b, days 1–4) served to assess the ability of the mice to acquire spatial information and learn the position of the hidden platform, which was the same each day. During this phase, the escape latency significantly decreased over time (LMM, *p* < 0.0001) without differences between genotypes (LMM, *p* = 0.99), indicating that both groups had the same spatial learning potential. On the fifth day, the platform was removed and all mice were tested for the ability to remember its previous position during a single probe trial (Fig. 3c–e). The memory retention, defined as the accuracy ratio (AR; time spent in the target quadrant multiplied by 3 and divided by the time spent in the others three quadrants), was significantly lower for the NURR1-KO mice compared to their WT littermates (Fig. 3c, t-test, *p* = 0.002). In particular, unlike WT mice which preferentially travelled in the target quadrant, NURR1-KO mice spent approximately the same time in each quadrant (median AR = 1.09). In addition, the latency to reach the position of the removed platform during the probe trial was similar for the two groups of mice (Fig. 3d, Mann–Whitney U test, *p* = 0.11), meaning that the capability to reach the target is not altered in the NURR1-KO mice. These findings led us to measure the path length after mice reached the target zone. An increased path length outside the target zone (Q^1^ + Q^2^ + Q^3^), after reaching the original location of the platform, would suggest an attempt to find a new location of the platform and thus an increased behavioral flexibility. On the contrary, longer path length in the target zone (Q^T^) would suggest a persistency, possibly related to an increased anxiety^32^. Here, NURR1-KO mice displayed a significantly longer path length outside the target zone compared with their WT littermates (Fig. 3e, *t*-test, *p* = 0.03).

Finally, mice underwent the reversal task (Fig. 3b, days 6–8). On day 6 the platform location was changed and the acquisition of a new memory was tested for three days. NURR1-KO mice showed a shorter latency to find the new platform compared to WT, especially in the sixth day (LMM, time effect: *p* < 0.0001, genotype effect: *p* = 0.06), further confirming their increased behavioral flexibility.

### NURR1-KO mice do not show a defect in social behavior and memory

Social behavior and social memory were evaluated using the three chamber sociability test (Fig. 3f–g). The analysis during the habituation phase did not highlight any innate preference for a chamber in either mice group (data not shown). Both groups displayed social behavior, defined as spending more time sniffing the mouse than sniffing the object (Fig. 3f, *t*-test, WT: *p* < 0.0001, NURR1-KO: *p* = 0.03). Furthermore, they exhibit a similar social memory, as they both spent more time sniffing the novel mouse than the familial one (Fig. 3g, *t*-test, WT: *p* = 0.001, NURR1-KO: *p* = 0.01).

### NURR1-KO mice do not show a deficit of the mDA TH + neurons number and DA levels

**Fig. 4:**
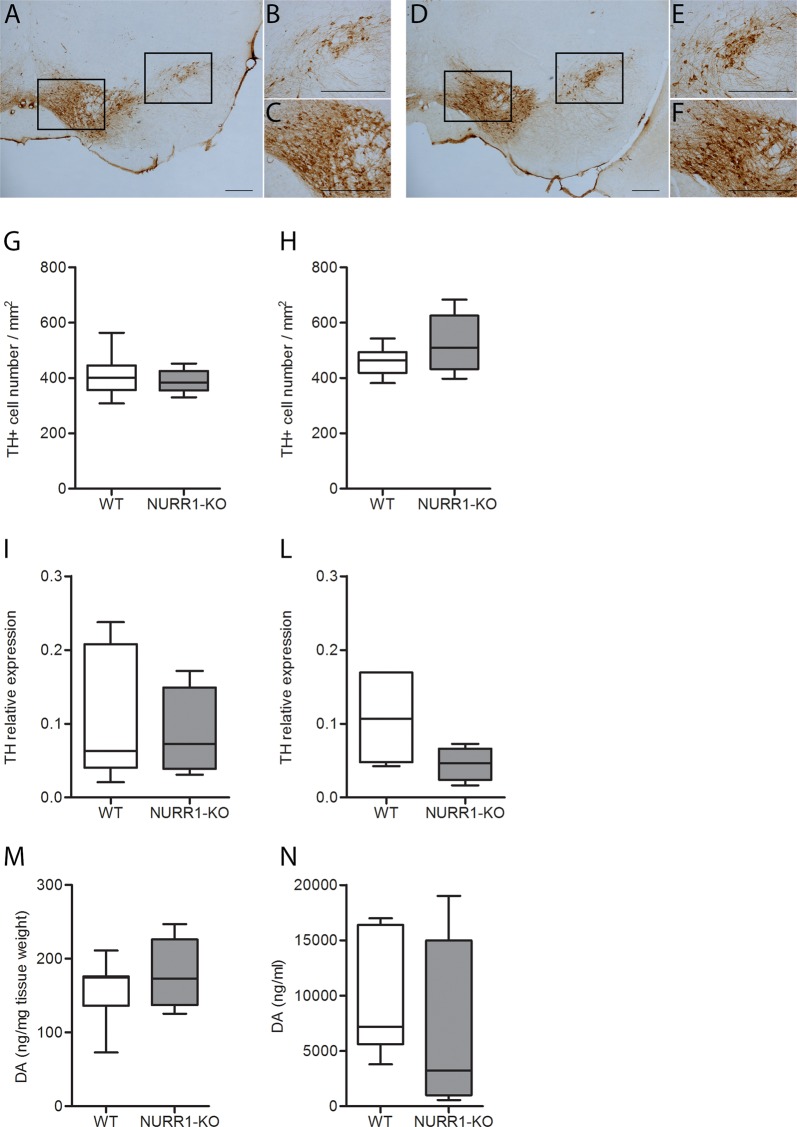
NURR1-KO mice do not show a deficit of the mDA TH + neurons and DA levels. Representative images of coronal brain sections of WT (**a–c**) and NURR1-KO (**d–f**) mice stained with anti-TH antibody. The sections highlight the substantia nigra (SN) (**a**, **b**, **d**, **e**) and ventral tegmental area (VTA) (**a**, **c**, **d**, **f**) regions as indicated by the black box. Calibration bars, 250 µm. **g**, **h** Quantitative analysis of the TH + cell number in SN (**g**) and VTA (**h**) of WT (*n* = 9) and NURR1-KO (*n* = 7) mice disclosed no differences between genotypes (*t*-test, in SN *p* = 0.49; in VTA *p* = 0.12). **i**, **l** Also the real-time RT-PCR analysis revealed that the gene expression level of TH in SN (**i**) and VTA (**l**) was not different between WT (*n* = 7) and NURR1-KO (*n* = 4) (*t*-test, in SN *p* = 0.92; in VTA *p* = 0.11). **m**, **n** DA quantification in brain (**m**) and in plasma (**n**) disclosed no differences between WT (*n* = 6) and NURR1-KO (*n* = 10) mice (*t*-test, in brain *p* = 0.64; in plasma *p* = 0.17)

NURR1 has a crucial role in controlling the functioning of the mDA cells, which modulate locomotion and impulsive behavior. mDA TH + cells were thus counted in the SN and VTA of WT and NURR1-KO mice (Fig. 4a–f). No differences emerged between genotypes in both SN (Fig. 4g, *t*-test, *p* = 0.49) and VTA (Fig. 4h, *t*-test, *p* = 0.12). Real-time RT-PCR analysis confirmed these data, as the gene expression level of TH in both SN (Fig. 4i, *t*-test, *p* = 0.92) and VTA (Fig. 4l, *t*-test, *p* = 0.11) was not significantly different between genotypes. Also, the analysis of DA levels in brain (Fig. 4m, *t*-test, *p* = 0.64) and plasma (Fig. 4n, *t*-test, *p* = 0.17) revealed no differences between genotypes.

### MPH rescues hyperactivity but not impulsivity of NURR1-KO mice

**Fig. 5:**
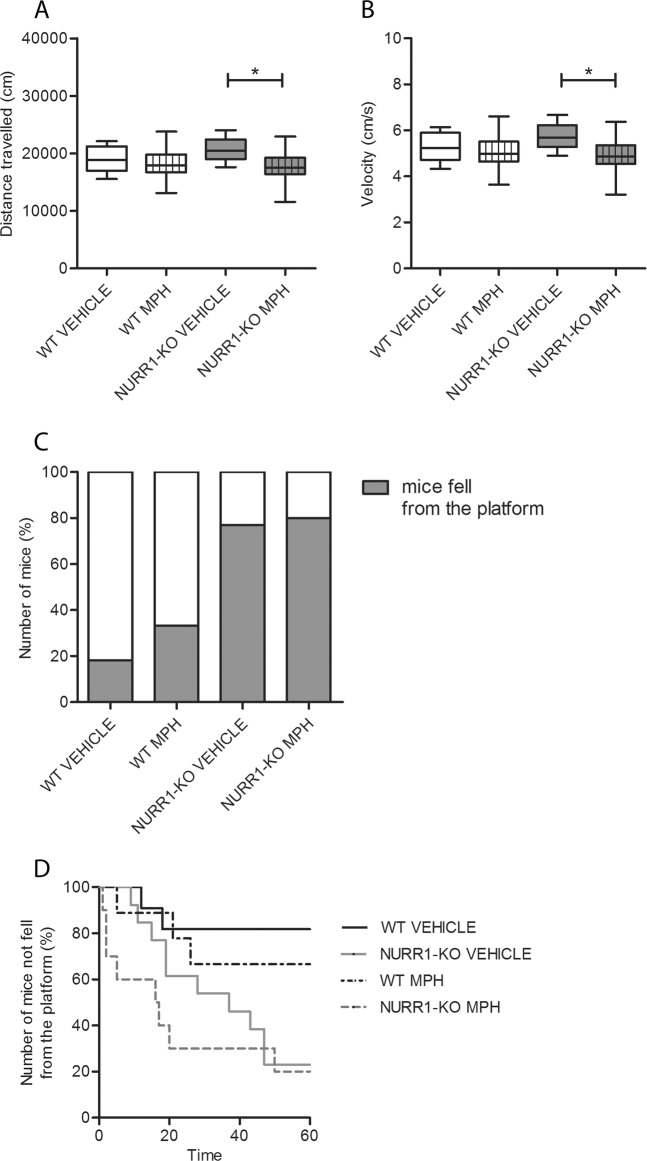
MPH rescues the altered locomotor activity of NURR1-KO mice but not their impulsive behavior. Both WT and NURR1-KO mice treated with vehicle (0.9% NaCl) or MPH (0.75 mg/kg) 1 h before were tested in the open field (OF, **a**, **b**) or cliff avoidance reaction (CAR, **c**, **d**) for 1 h. **a**, **b** The analysis disclosed that NURR1-KO mice treated with MPH show a rescue in their altered locomotor activity, in terms of distance travelled (**a**) (Kruskal–Wallis with Dunn’s post hoc test, df = 3, *p* = 0.03) and velocity (**b**) (Kruskal–Wallis with Dunn’s post hoc test, *p* < 0.0001, df = 3, *p* = 0.03). The NURR1-KO mice treated with MPH perform similarly to WT mice treated with vehicle and MPH (Kruskal–Wallis with Dunn’s post hoc test, vehicle distance travelled: *p* = 0.33, velocity: *p* = 0.29; MPH distance travelled: *p* = 0.39, velocity: *p* = 0.38). Number of mice tested for OF: 13 WT vehicle, 14 NURR1-KO vehicle, 9 WT MPH, 10 NURR1-KO MPH. **c** The analysis of CAR test displayed that NURR1-KO mice treated with MPH did not show a rescue in their impulsive behavior (Fisher exact test, *p* = 1). Gray box indicates the percentage of mice which fell from the platform. **d** Also the time course of CAR impairment in WT and NURR1-KO mice after the MPH administration was reported. Number of mice tested for CAR 11 WT vehicle, 13 NURR1-KO vehicle, 9 WT MPH, 10 NURR1-KO MPH

The effects of MPH treatment on locomotor activity and impulsive behavior was examined in a separate cohort of mice. WT and NURR1-KO mice, treated with vehicle (0.9% NaCl) or MPH (0.75 mg/kg), underwent OF or CAR tests 1 h after drug/vehicle administration (Fig. 5). NURR1-KO mice treated with MPH showed a decreased locomotor activity in comparison to their NURR1-KO vehicle treated littermates, in terms of both distance travelled (Fig. 5a, Kruskal–Wallis with Dunn’s post hoc test, df = 3, *p* = 0.03) and velocity (Fig. 5b, Kruskal–Wallis with Dunn’s post hoc test, df = 3, *p* = 0.03) at the OF test. Particularly, NURR1-KO mice treated with MPH performed similarly to WT mice treated with vehicle (Kruskal–Wallis with Dunn’s post hoc test, df = 3, distance travelled: *p* = 0.33, velocity: *p* = 0.29) or MPH (Kruskal–Wallis with Dunn’s post hoc test, df = 3, distance travelled: *p* = 0.39, velocity: *p* = 0.38). On the other hand, the MPH treatment did not rescue the impulsive behavior of the NURR1-KO mice at the CAR test. In fact, 80% (8 out of 10) of the NURR1-KO mice treated with MPH fell from the platform compared to 77% (10 out of 13) of their littermates with the same genotype treated with vehicle (Fig. 5c, Fisher exact test, *p* = 1). Meanwhile, 33.3% (three out of nine) of the WT mice treated with MPH and 18% (two out of 11) of the WT mice treated with vehicle fell from the platform, indicating no treatment-effects on impulsivity of WT mice.

## Discussion

The transcription factor NURR1 regulates genes of the DA signaling pathway^2,4,5^ and is essential for the mDA development^7,37^. This is probably due to its ability to not only regulate genes of the DA signaling pathway^2,4,5^, but also to protect mDA against inflammation-induced damage^6,7,37^. Given its functions, it play a critical role in DA-associated brain disorders, such as schizophrenia and PD^8–11^.

Animal models able to recapitulate the key phenotypes of neurological disorders are crucial to understand the biological basis of the diseases and to test the clinical efficacy of potential therapeutic agents, as reported in their face, construct and predictive validity. The NURR1-KO mouse was suggested as a model for schizophrenia^21^ and PD^20^, but further studies highlighted that it only exhibits a restricted behavioral phenotype of these diseases^38^. Given its increased spontaneous locomotor activity^19–22^ the NURR1-KO mouse could be proposed as well as a model of ADHD. However, to date a complete characterization of this model is still lacking.

In the present study, we performed a comprehensive analysis of the behavioral phenotype of the NURR1-KO mice. The use of a wide-ranging test battery allowed us to demonstrate the presence of two main ADHD symptoms, in the absence of other non-specific behavioral alterations. First, we confirmed their increased spontaneous locomotor activity at the OF test in a novel environment. This result is reasonably reliable, considering that this phenotype has been observed in three other independent laboratories^19–22^ using three different NURR1-KO models^1,2,39^. Second, for the first time to our knowledge, we reported an impulsive behavior in the NURR1-KO mice at the CAR test. Third, we have not evaluated their attention. However, in previous studies, the NURR1-KO mice did not exhibit an impairment in attention using the multiple-choice visual discrimination and the latent inhibition tests^22,23^. It is known that these tests require long-term experimental procedures, including food/water restriction, which are highly stressful and could affect the attentional function of the animals^40^. Here, we tested only male mice to avoid confounding results related to sex. It is noteworthy that ADHD seems to affect more boys than girls^13^ and that the behavioral problems of ADHD boys seems to differ from those of ADHD girls^41^. Particularly, the primary deficit in boys may be described as hyperactivity and impulsiveness without attention problems that instead affects the girls. Accordingly, our demonstration that NURR1-KO mice show hyperactivity and impulsivity without attention problems as reported by^23^ make it a valid model for ADHD (face validity).

Fourth, no alterations emerged in their motor coordination at the rota-rod test. This is in agreement with reports highlighting no alterations in the performance of adult (i.e. between 2 and 10 months of age) NURR1-KO mice compared to their WT littermates^20^. On the contrary, the rota-rod performance have been demonstrated to decrease in old NURR1-KO mice (i.e. from 15 months of age), suggesting motor impairment as an elderly PD defect. Fifth, we excluded an anxiety-like behavior at the EPM test. Sixth, we corroborate previous data reporting no abnormalities in spatial recognition memory and learning and social interaction and recognition^22^ using different experimental approach, such as Morris water maze and three-chambered sociability test instead of T-maze and Y-maze tests, respectively.

Besides performing behavioral tests, we also evaluated the number of TH-expressing mDA and the TH transcript level in SN and VTA, obtaining no differences between NURR1-KO and WT mice. On this matter, contrasting results can be found in the literature. According to our data, Jiang and Kummari found no alterations in the number of TH-expressing mDA in the SN of adult NURR1-KO mice^20,42^. On the contrary, Vuillermot and colleagues reported a reduction of these mDA in NURR1-KO mice of the same age^23^. Notably, mice with a different genetic background were used. In particular, in Jiang’s and our study NURR1-KO mice created by Saucedo-Cardenas and colleagues^2^ were tested at the same age^20^. Also, DA level of NURR1-KO mice reveled no differences between genotypes. These data are in contrast with previous results reporting a decreases level of DA in whole brain^19^. However, they analyzed younger mice with different genetic background^19^.

Finally, we found that acute treatment with MPH, a stimulant drug currently used to correct the ADHD symptoms, is able to partially restore the behavioral phenotype of the NURR1-KO mice. Interestingly, MPH is able to rescue the hyperactive behavior but not the impulsive one. Considering that the treatment of psychiatric diseases involves the chronic administration of drugs, the results obtained from acute treatment can be considered an excellent starting point. Overall, our study suggests that the NURR1 deficient male mouse may be a satisfactory model to study some behavioral phenotypes such as hyperactivity and impulsivity characteristic of the human hyperactive-impulsive subtypes of ADHD according to the DSM-V. Various animal models, such as the spontaneous hypertensive rats (SHR)^43^, the neonatal 6-hydroxydopamine (6-OHDA) rats^44^ and DAT-KO^45^ mice are useful tool to study at least in part the heterogeneous aspects of ADHD symptomatology and neurobiology. Notably, in these animal models the hyperactivity and the impulsive/attentive defects are evaluated using the OF and the multiple-choice visual discrimination and latent inhibition tests, respectively. Here, we evaluated the face validity of the NURR1-KO mice through assessing the spontaneous activity in the OF and impulsivity in an easier investigation such as CAR. Specifically, this well-demonstrated impulsive behavior represent a suitable model to study impulsivity without long experimental training and food/water deprivation that could stress the animals. Although the NURR1-KO mouse model failed to show inattention^23^, this does not make it any less valuable, since in humans attention deficit seems to especially affect girls while hyperactivity and impulsiveness affect boys the most^13,41^.

## Supplementary information

Materials and Methods S

Table S1

Figure S1

Figure legend S1

## References

[1] Zetterstrom R. H., et al. Dopamine neuron agenesis in Nurr1-deficient mice. *Science* **276**, 248–251 (1997). 10.1126/science.276.5310.248 9092472

[2] Saucedo-Cardenas O (1998). Nurr1 is essential for the induction of the dopaminergic phenotype and the survival of ventral mesencephalic late dopaminergic precursor neurons. Proc. Natl Acad. Sci. USA.

[3] Solomin L., Arvidsson M., Olson L., Perlmann T. Fate of mesencephalic AHD2-expressing dopamine progenitor cells in Nurr1 mutant mice. *Exp. Cell. Res.* **746**, 737–746 (1999). 10.1006/excr.1999.4691 10585298

[4] Kadkhodaei B., Ito T., Joodmardi E., Mattsson B., Rouillard C., Carta M., Muramatsu S.-I., Sumi-Ichinose C., Nomura T., Metzger D., Chambon P., Lindqvist E., Larsson N.-G., Olson L., Bjorklund A., Ichinose H., Perlmann T. (2009). Nurr1 Is Required for Maintenance of Maturing and Adult Midbrain Dopamine Neurons. Journal of Neuroscience.

[5] Smidt M. P. and Burbach J. P. H. How to make a mesodiencephalic dopaminergic neuron. **8**, 21–32 (2008). 10.1038/nrn2039 17180160

[6] Saijo K (2009). A Nurr1/CoREST pathway in microglia and astrocytes protects dopaminergic neurons from inflammation-induced death. Cell.

[7] Kadkhodaei B (2013). Transcription factor Nurr1 maintains fiber integrity and nuclear-encoded mitochondrial gene expression in dopamine neurons. Proc. Natl Acad. Sci. USA.

[8] Jankovic J., Chen S., Le W.D. (2005). The role of Nurr1 in the development of dopaminergic neurons and Parkinson's disease. Progress in Neurobiology.

[9] Montarolo Francesca, Perga Simona, Martire Serena, Navone Désirée Nicole, Marchet Alberto, Leotta Daniela, Bertolotto Antonio (2016). Altered NR4A Subfamily Gene Expression Level in Peripheral Blood of Parkinson’s and Alzheimer’s Disease Patients. Neurotoxicity Research.

[10] Buervenich S., et al. NURR1 Mutations in Cases of Schizophrenia and Manic-Depressive Disorder. **813**, 808–813 Am. *J. Med. Genet.* (2000). 10.1002/1096-8628(20001204)96:6<808::aid-ajmg23>3.0.co;2-e 11121187

[11] Xing Guoqiang, Zhang Lei, Russell Shani, Post Robert (2006). Reduction of dopamine-related transcription factors Nurr1 and NGFI-B in the prefrontal cortex in schizophrenia and bipolar disorders. Schizophrenia Research.

[12] BARKLEY RUSSELL A. (2004). Adolescents with Attention-Deficit/Hyperactivity Disorder: An Overview of Empirically Based Treatments. Journal of Psychiatric Practice.

[13] Biederman Joseph, Faraone Stephen V (2005). Attention-deficit hyperactivity disorder. The Lancet.

[14] Wilens TE (2008). Effects of methylphenidate on the catecholaminergic system in attention-deficit/hyperactivity disorder. J. Clin. Psychopharmacol..

[15] Razoki Bashar (2018). Neurofeedback versus psychostimulants in the treatment of children and adolescents with attention-deficit/hyperactivity disorder: a systematic review. Neuropsychiatric Disease and Treatment.

[16] Bäckman C, You ZB, Perlmann T, Hoffer BJ (2003). Elevated locomotor activity without altered striatal dopamine contents in Nurr1 heterozygous mice after acute exposure to methamphetamine. Behav. Brain Res..

[17] Zayats T., et al., Genome-wide analysis of attention deficit hyperactivity disorder in Norway. *Plos One* **10**, 1–17 (2015). 10.1371/journal.pone.0122501 PMC4395400 25875332

[18] Smith KM, Bauer L, Fischer M, Barkley R, Navia BA (2005). Identification and characterization of human NR4A2 polymorphisms in attention deficit hyperactivity disorder. Am. J. Med Genet. B Neuropsychiatr. Genet..

[19] Eells JB, Lipska BK, Yeung SK, Misler JA, Nikodem VM (2002). Nurr1-null heterozygous mice have reduced mesolimbic and mesocortical dopamine levels and increased stress-induced locomotor activity. Behav. Brain Res..

[20] Jiang C (2005). Age-dependent dopaminergic dysfunction in Nurr1 knockout mice. Exp. Neurol..

[21] Rojas P, Joodmardi E, Hong Y, Perlmann T, Ogren SO (2007). Adult mice with reduced Nurr1 expression: an animal model for schizophrenia. Mol. Psychiatry.

[22] Vuillermot S (2011). Schizophrenia-relevant behaviors in a genetic mouse model of constitutive Nurr1 deficiency. Genes, Brain Behav..

[23] Vuillermot S (2012). Prenatal immune activation interacts with genetic Nurr1 deficiency in the development of attentional impairments. J. Neurosci..

[24] Cortese S (2015). Safety of methylphenidate and atomoxetine in children with attention-deficit/hyperactivity disorder (ADHD): data from the italian national ADHD Registry. CNS Drugs.

[25] Giedd JN, Blumenthal J, Molloy E, Castellanos FX (2001). Brain imaging of attention deficit/hyperactivity disorder. Ann. N. Y Acad. Sci..

[26] Gainetdinov RR, Mohn AR, Bohn LM, Caron MG (2001). Glutamatergic modulation of hyperactivity in mice lacking the dopamine transporter. Proc. Natl Acad. Sci. USA.

[27] Yamashita Motoyasu, Sakakibara Yasufumi, Hall F. Scott, Numachi Yohtaro, Yoshida Sumiko, Kobayashi Hideaki, Uchiumi Osamu, Uhl George R., Kasahara Yoshiyuki, Sora Ichiro (2013). Impaired cliff avoidance reaction in dopamine transporter knockout mice. Psychopharmacology.

[28] Saucedo-Cardenas O, Kardon R, Ediger TR, Lydon JP, Conneely OM (1997). Cloning and structural organization of the gene encoding the murine nuclear receptor transcription factor, NURR1. Gene.

[29] Hoxha E (2013). Molecular and cellular neuroscience motor dysfunction and cerebellar Purkinje cell fi ring impairment in Ebf2 null mice. Mol. Cell Neurosci..

[30] Pellegrino R. M., et al., Transferrin receptor 2 dependent alterations of brain iron metabolism affect anxiety circuits in the mouse. *Sci. Rep*. **6**, 30725 (2016). 10.1038/srep30725 PMC4967901 27477597

[31] Montarolo F, Parolisi R, Hoxha E, Boda E, Tempia F (2013). Early enriched environment exposure protects spatial memory and accelerates amyloid plaque formation in APPSwe/PS1L166P mice. PLoS ONE.

[32] Longo A., Oberto A., Serra M., Eva C. NPY-Y1 coexpressed with NPY-Y5 receptors modulate. *Genes Brain Behav.* **14**, 534–542 (2015). 10.1111/gbb.12232 26178014

[33] Koenig J, Cosquer B, Cassel J (2008). Activation of septal 5-HT1A receptors alters spatial memory encoding, interferes with consolidation, but does not affect retrieval in rats subjected to a water-maze task. Hippocampus.

[34] Yang M., Silverman J. L., Crawley J. N. Automated three-chambered social approach task for mice. *Curr. Protoc. Neurosci*. **8**, 1–16 (2011). 10.1002/0471142301.ns0826s56 PMC4904775 21732314

[35] Marcinnò A (2019). Emerging roles of Fgf14 in behavioral control. Behav. Brain Res..

[36] Balcioglu Aygul, Ren Jia-Qian, McCarthy Deirdre, Spencer Thomas J., Biederman Joseph, Bhide Pradeep G. (2009). Plasma and brain concentrations of oral therapeutic doses of methylphenidate and their impact on brain monoamine content in mice. Neuropharmacology.

[37] Bensinger SJ, Tontonoz P (2009). A Nurr1 Pathway for Neuroprotection. Cell.

[38] Vuillermot S, Weber L, Feldon J, Meyer U (2010). A longitudinal examination of the neurodevelopmental impact of prenatal immune activation in mice reveals primary defects in dopaminergic development relevant to schizophrenia. J. Neurosci..

[39] Castillo SO (1998). Dopamine biosynthesis is selectively abolished in substantia nigra/ventral tegmental area but not in hypothalamic neurons in mice with targeted disruption of the Nurr1 gene. Mol. Cell Neurosci..

[40] Tucci V, Hardy A, Nolan PM (2006). A comparison of physiological and behavioural parameters in C57BL/6J mice undergoing food or water restriction regimes. Behav. Brain Res..

[41] Lahey BB (2007). Are there sex differences in the predictive validity of DSM–IV ADHD among younger children?. J. Clin. Child Adolesc. Psychol..

[42] Kummari E, Guo-Ross S, Eells JB (2017). Region specific effects of aging and the nurr1-null heterozygous genotype on dopamine neurotransmission. Neurochem. Neuropharmacol..

[43] Sagvolden T, Johansen EB (2012). Rat models of ADHD. Curr. Top. Behav. Neurosci..

[44] Bouchatta O (2018). Neonatal 6-OHDA lesion model in mouse induces attention-deficit/ hyperactivity disorder (ADHD)-like behaviour. Sci. Rep..

[45] Trinh JV, Nehrenberg DL, Jacobsen JP, Caron MG, Wetsel WC (2003). Differential psychostimulant-induced activation of neural circuits in dopamine transporter knockout and wild type mice. Neuroscience.

