# Supplementary material for: NURR1 deficiency is associated to ADHD-like phenotypes in mice
Source: Transl Psychiatry. 2019 Aug 27;9:207. doi: 10.1038/s41398-019-0544-0 (PMC6712038; doi:10.1038/s41398-019-0544-0)
Supplement: Supplementary file 1 — Materials and Methods S [file 41398_2019_544_MOESM1_ESM.docx]

**Supplementary material**

**Material and Methods**

**Morris water maze test**

The maze was located in a room, where the mouse has never been, with numerous extra-maze cues visible to the mice during testing. It consisted of a circular pool (diameter 140 cm; height 40 cm) filled with water at 25°C to avoid hypothermia added with milk powder to hide a submerged platform. A small escape platform made of transparent plastic (diameter 15 cm) was placed in the center of one quadrant 35 cm far from the side-wall of the pool at a fixed position and was hidden 1 cm beneath the water surface. The acquisition phase (days 1-4) consisted of 4 training days (days 1-4) with four trials per day with a 15 min inter-trial interval. Four points equally spaced along the circumference of the pool (i.e. north, south, west, east) served as the starting position, which was randomized across the four trials each day. For each trial, the mouse was released from the side of the pool, facing the wall. The time spent to reach platform (escape latency) was video-recorded for each animal. During first trial of the first day (when the mice knew anything about the spatial location of the platform) also swimming velocity was measured to evaluate locomotor skills and motivation. If an animal did not reach the platform within 90 sesconds (s), it was guided to the platform, where it was allowed to rest for 30 s, before returning to the home cage. Between trials, mice were kept dry in a cage filled with paper towels. On day 5, a probe trial was performed to assess spatial memory after a 24 h delay since the last acquisition trial. In the probe trial, the platform was removed from the maze and the animals were allowed to swim freely for 60 s. The time spent in each quadrant (quadrant 1, Q^1^; quadrant 2, Q^2^; quadrant 3, Q^3^ and target quadrant, Q^T^ , in which the escape platform was located during the acquisition phase) was recorded. The primary retention measure was the accuracy ratio (AR) (33) calculated as follows: time spent in the target quadrant (TQ^T^) multiplied by 3 and divided by the time spent in the other three quadrants (TQ^1^ + TQ^2^ + TQ^3^). An AR of 1 corresponds to chance level, indicating no preference for Q^T^ relative to the other quadrants. An AR of 2 corresponds to 24 s spent in Q^T^, while an accuracy ratio of 3 corresponds to 30 s in Q^T^. In the probe trial, also the latency to reach the location of the removed platform and path length after the target zone was reached were assessed. On day 6, 7 and 8 a reversal training task was carried out as reported by (32). This involved changing the location of the escape platform in the opposite quadrant of the pool compared to its position on the acquisition phase. Mice received four trials per day with a 15 min inter-trial interval and were given 60 s to find the hidden platform. The escape latencies were video-recorded.

**Three-chambered sociability test**

The apparatus, a custom-made clear Plexiglas box partitioned into three chambers of equal size (20 × 40.5 × 22 cm), was designed as previously described 34-35). The test was divided in three phases, habituation, social behavior and social memory.

During habituation, the mouse was confined in the middle chamber for 10 min. After this phase the doors were opened and the mouse was permitted to freely move in all chambers for other 10 min. During social behavior, the mouse was confined again in the middle chamber. An inverted empty wire cup (wire pencil cup, Galaxy Cup, Kitchen Plus, http://www.kitchen-plus.com) and a wire cup containing a stranger mouse were placed into left and right chambers. The doors were re-opened and the mouse was allowed to explore all chambers for other 10 minutes (min). The comparison of the time spent to explore stranger mouse vs. empty wire cup (object) indicated the sociability of animals. Time explored was defined as the time in which the mouse’s nose was placed closed to the wire cup containing objects or mouse to sniff. During social memory, the mouse was confined again in the middle chamber. The inverted empty wire cup was substituted with a wire cup containing a novel mouse. The stranger mouse used during social behavior remained confined in the wire cup (familial mouse). The doors were re-opened and the mouse was allowed to explore all chambers for other 10 min. The comparison of the time spent to explore novel mouse vs. familial mouse indicated the social memory of animals.

Stranger mice (familial and novel) were housed in the same facility but had no prior contact with test mice. They were trained for two sessions of 15 min a day before the test. The observer remained in the room and only mice that at the end of 15 min did not grip to the wire cup were chosen. The stranger mice (familial and novel) were adults mice.

**Systolic blood pressure and heart rate measurement**

Blood pressure and heart rate were recorded using a noninvasive tail cuff-based technique (BP-2000 Series II, Blood Pressure Analysis System, Visitech Systems, Apex, NC). Prior to measurements, the animals were placed into the habituation room for 30 min for to ensure mice adaptation to the procedure. Parameters were recorded in a proper environment (RT, lightning and noise-free atmosphere). Conscious adults male NURR1-KO (n=10) and WT mice (n=6) (see Table S1) underwent 5 cycle measurement of systolic blood pressure and heart rate. Data are expressed as median and range.
