# Supplementary material for: NURR1 deficiency is associated to ADHD-like phenotypes in mice
Source: Transl Psychiatry. 2019 Aug 27;9:207. doi: 10.1038/s41398-019-0544-0 (PMC6712038; doi:10.1038/s41398-019-0544-0)
Supplement: Supplementary file 2 — Table S1 [file 41398_2019_544_MOESM2_ESM.docx]

**Table S1. Group size, sequence of testing and time spent for each test**

|  | **WT**  **(N)** | **NURR1-KO**  **(N)** | **EXPERIMENTS** |
| --- | --- | --- | --- |
| **COHORT 1** | 9 | 7 | Three months old mice (day 0):  Day 0 OF;  Day 7 CAR;  Day 14-16 Rota-rod (3 days);  Day 23 EPM;  Day 30-37 Morris water maze (8 days) ;  Day 44 Three chamber sociability;  Day 51 sacrifice for histological procedures (5 months old mice). |
| **COHORT 2** | 7 | 4 | Four months old mice:  Sacrifice for biomolecular analysis. |
| **COHORT 3** | 6 | 10 | Four months old mice (day 0):  Day 0 Blood pressure and heart rate measurement;  Day 7 Sacrifice for DA measurement. |
| **COHORT 4** | 22  (13 vehicle; 9 MPH) | 24  (14 vehicle; 10 MPH) | Four months old mice:  OF. |
| **COHORT 5** | 20  (11 vehicle; 9 MPH) | 23  (13 vehicle; 10 MPH) | Four months old mice:  CAR. |
| **TOTAL** | 64 | 68 |  |

WT; wild type, KO; knockout, N; number of animals, Open field; OF, Cliff avoidance reaction; CAR, Elevated plus maze; EPM, Methylphenidate; MPH.
