# Supplementary figures and images for: NURR1 deficiency is associated to ADHD-like phenotypes in mice
Source: Transl Psychiatry. 2019 Aug 27;9:207. doi: 10.1038/s41398-019-0544-0 (PMC6712038; doi:10.1038/s41398-019-0544-0)

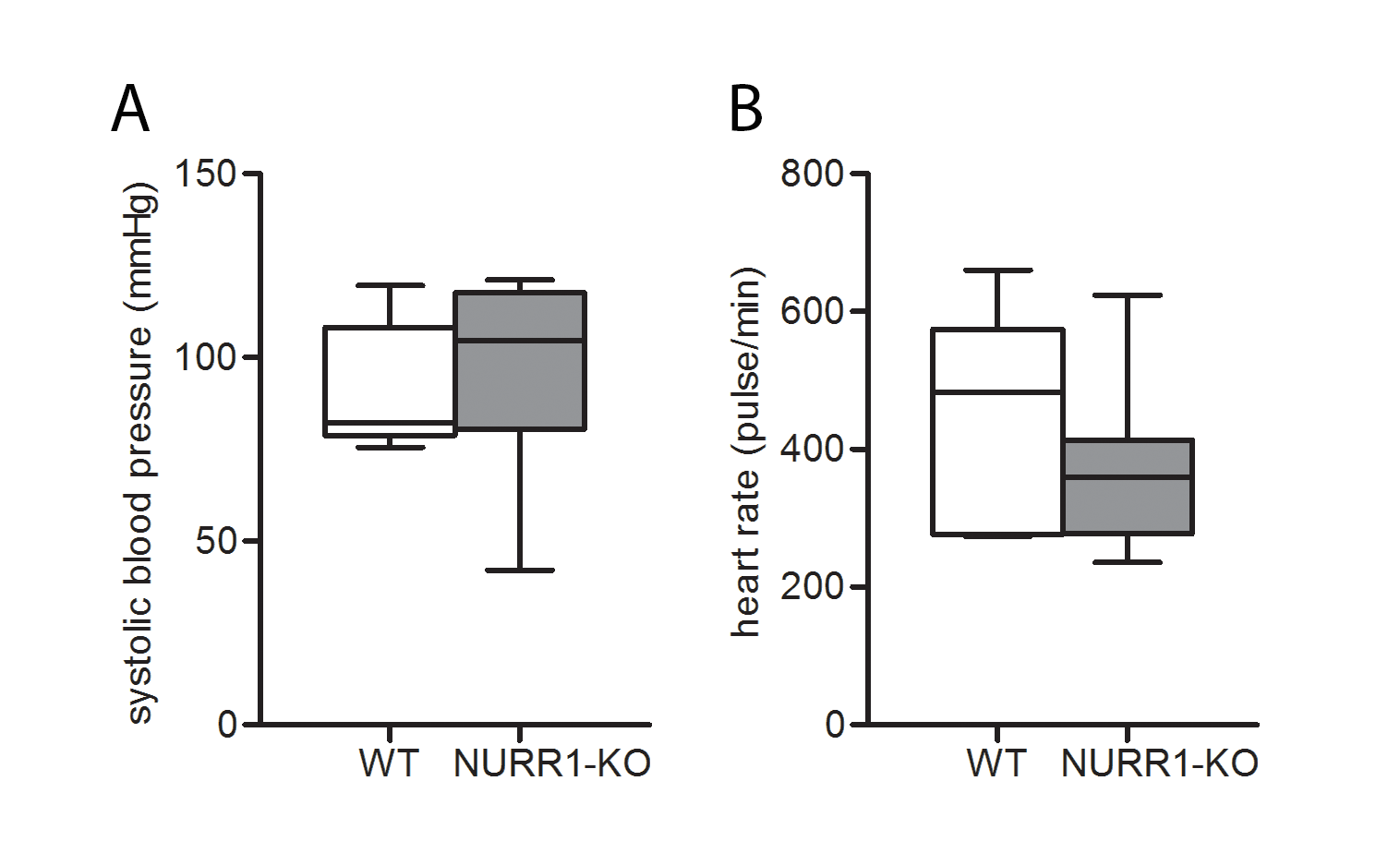

Supplement: Supplementary file 3 — Figure S1 [file 41398_2019_544_MOESM3_ESM.tif]
