# Supplementary material for: NURR1 deficiency is associated to ADHD-like phenotypes in mice
Source: Transl Psychiatry. 2019 Aug 27;9:207. doi: 10.1038/s41398-019-0544-0 (PMC6712038; doi:10.1038/s41398-019-0544-0)
Supplement: Supplementary file 4 — Figure legend S1 [file 41398_2019_544_MOESM4_ESM.docx]

**Figure legends**

**Figure S1. NURR1-KO mice do not show a defect in blood pressure and heart rate.** Both WT (n=6) and NURR1-KO (n=10) were tested for systolic blood pressure (A) and heart rate (B). No significant differences between the groups emerged (Mann–Whitney U test, blood pressure p=0.44; heart rate p=0.44).
